# The Role of the Phosphatidylinositol 4-Kinase PI4KA in Hepatitis C Virus-Induced Host Membrane Rearrangement

**DOI:** 10.1371/journal.pone.0026300

**Published:** 2011-10-12

**Authors:** Andrew W. Tai, Shadi Salloum

**Affiliations:** 1 Division of Gastroenterology, Department of Internal Medicine, University of Michigan, Ann Arbor, Michigan, United States of America; 2 Division of Gastroenterology, Department of Internal Medicine, Ann Arbor Veterans Administration Health System, Ann Arbor, Michigan, United States of America; University of Cambridge, United Kingdom

## Abstract

**Background:**

Hepatitis C virus (HCV), like other positive-sense RNA viruses, replicates on an altered host membrane compartment that has been called the “membranous web.” The mechanisms by which the membranous web are formed from cellular membranes are poorly understood. Several recent RNA interference screens have demonstrated a critical role for the host phosphatidylinositol 4-kinase PI4KA in HCV replication. We have sought to define the function of PI4KA in viral replication.

**Methodology/Principal Findings:**

Using a nonreplicative model of membranous web formation, we show that PI4KA silencing leads to aberrant web morphology. Furthermore, we find that PI4KA and its product, phosphatidylinositol 4-phosphate, are enriched on membranous webs and that PI4KA is found in association with NS5A in HCV-infected cells. While the related lipid kinase PI4KB also appears to support HCV replication, it does not interact with NS5A. Silencing of PI4KB does not overtly impair membranous web morphology or phosphatidylinositol 4-phosphate enrichment at webs, suggesting that it acts at a different point in viral replication. Finally, we demonstrate that the aberrant webs induced by PI4KA silencing require the activity of the viral NS3-4A serine protease but not integrity of the host secretory pathway.

**Conclusions/Significance:**

PI4KA is necessary for the local enrichment of PI 4-phosphate at the HCV membranous web and for the generation of morphologically normal webs. We also show that nonreplicative systems of web formation can be used to order molecular events that drive web assembly.

## Introduction

Hepatitis C virus (HCV) is a positive-sense ssRNA virus that is estimated to chronically infect as many as 3% of the world's population, of whom up to 30% will progress to cirrhosis. As a result, HCV-related liver disease (principally liver failure and hepatocellular carcinoma) is the leading indication for liver transplantation worldwide. Recent drug development strategies to combat HCV infection have largely focused on the viral NS3-4A serine protease and NS5B RNA polymerase, although viral resistance remains a concern due to the error-prone nature of the viral polymerase [1]. We have instead focused on defining the host cofactors that support the viral lifecycle, as blocking cellular cofactors may impose a higher barrier to resistance and may permit the targeting of multiple steps in the viral lifecycle.

A number of RNAi screens for host cofactors of HCV replication have identified a critical role for the phosphatidylinositol (PI) 4-kinase PI4KA in HCV replication e.g. [2]–[6]. Four different mammalian PI 4-kinases have been identified (reviewed in [7]), which all catalyze the conversion of PI to PI 4-phosphate. PI(4)P is believed to exert its functions through the binding of a number of effector proteins, including the coat adaptor AP-1 [8] and lipid transfer proteins such as OSBP1 and CERT (reviewed in [9]). Intriguingly, the related PI 4-kinase PI4KB has recently been shown to be required for enterovirus replication [10], suggesting a common dependence of at least some positive-sense ssRNA viruses on host PI(4)P metabolism. In particular, all positive-sense ssRNA viruses studied to date replicate on altered cellular membrane compartments, which in the case of HCV has been termed the “membranous web” [11].

Little is known about the mechanisms that direct HCV membranous web formation. While the membranous web is generally believed to be derived from the host endoplasmic reticulum (ER), it is a detergent-resistant membrane [12] and has also been shown to be associated with early endocytic markers such as Rab5 [13], suggesting that the membranous web is highly modified from its membrane(s) of origin. We lack a precise understanding of the molecular events that transform the host ER into the membranous web, in part because intermediate structures in web formation have not been characterized.

We have sought to determine the mechanisms by which PI4KA and PI(4)P support HCV replication. Because HCV polyprotein translation is coupled to RNA replication, silencing of essential host cofactors such as PI4KA leads to the rapid loss of HCV polyprotein translation in authentic replication systems (replicons or infectious virus). As a result, blocks in membranous web assembly are difficult or impossible to identify in replication-dependent HCV expression systems but may be recognizable in nonreplicative HCV expression systems. We had previously found that PI4KA silencing in U2-OS osteosarcoma cells inducibly expressing a full-length HCV polyprotein [14] led to abnormal NS5A-positive membrane clusters [2]. However, U2-OS cells do not support efficient HCV replication, and so we sought to establish a replication-independent HCV expression system in the more physiologically relevant Huh7 hepatoma cell line. Using a T7 RNA polymerase-driven system of HCV polyprotein expression, we show that PI4KA is required for the formation of membranous webs, and that PI4KA and its product PI(4)P are enriched at HCV replication sites. We also identify a potential intermediate of web formation that is generated in the absence of PI4KA activity; furthermore, we show that this intermediate requires viral NS3-4A serine protease activity but not brefeldin A-sensitive host factors, which are known to support viral replication. In principle, the methodologies used in this work can be applied to study other events in HCV membranous web formation.

## Materials and Methods

### Reagents

Antibodies used included those directed against annexin A2 (rabbit polyclonal, Santa Cruz Biotechnology, Santa Cruz, CA), the FLAG epitope tag (mouse monoclonal clone M2; Sigma-Aldrich, St. Louis, MO), myc tag (mouse monoclonal clone 9E10; Sigma-Aldrich), PI4KA (rabbit polyclonal, Cell Signaling, Danvers, MA), PI4KB (rabbit polyclonal, Abcam, Cambridge, MA), PI(4)P (mouse IgM monoclonal, Echelon, Salt Lake City, UT), HCV NS5A (mouse monoclonal clone 9E10; Dr. Charles Rice, Rockefeller University, New York, NY), HCV NS3 (mouse monoclonal, ViroGen, Watertown, MA), and HCV NS4B (mouse monoclonal, Abcam).

### Cell lines and cell culture

Huh7.5.1 cells [15] were grown in Dulbecco's modified Eagle's medium (DMEM) supplemented with 10% fetal bovine serum (FBS), nonessential amino acids, 100 U/mL of penicillin, and 100 µg/mL of streptomycin. OR6 cells containing a full-length genotype 1b HCV replicon [16] and SGR-JFH1 genotype 2a subgenomic replicon cells [17] were grown in the same medium with the addition of 400 µg/mL G418.

### Expression of HCV nonstructural proteins using T7 RNA polymerase

The JFH1 sequence from NS3 through NS5B, which is necessary and sufficient for membranous web formation and viral RNA replication, was subcloned from the pSGR-JFH1 subgenomic replicon construct [17] into the pTM1 vector [18] in two steps. The pTM1 vector was designed for T7 RNA polymerase-driven protein expression and contains a T7 promoter and EMCV IRES upstream of a multiple-cloning site and T7 transcriptional terminator. In the first step, the NcoI-NcoI fragment from pSGR-JFH1 was ligated into the NcoI restriction site in pTM1 at the immediate 3′ end of the EMCV IRES. In the second step, the SpeI-XbaI fragment from pSGR-JFH1 or from pSGR-JFH1(NS5A-GFP) was ligated into SpeI-digested pTM1 vector containing the NcoI-NcoI fragment to generate pTM1(NS3-5B) and pTM1(NS3-5B/GFP), respectively. The correct orientations of the NcoI-NcoI and SpeI-XbaI fragments were confirmed by restriction enzyme mapping and by sequencing of the termini. To construct pSGR-JFH1(NS5A-GFP), we amplified the EGFP sequence with flanking MluI restriction sites (primers in Table 1) and ligated it into the Jc1/NS5A-MluI construct described below. The SpeI-XbaI fragment from Jc1/NS5A-MluI was ligated into SpeI/XbaI digested pSGR-JFH1. The orientation of the fragment was confirmed by restriction enzyme digestion. pSGR-JFH1 Rz/T7ter was constructed by fusing the antigenomic hepatitis D virus ribozyme to the end of the HCV 3′NTR using overlap extension PCR. The T7 terminator sequence was introduced immediately downstream of the ribozyme sequence by ligating an oligonucleotide linker into the XbaI restriction enzyme site.

**Table 1:** Primers used for DNA construct preparation.

Construct	Sequence
pBabe-puro (T7 RNA polymerase)	Forward: 5′-TTTAAA**GGATCC**GCCACCATGAACACGATTAACATCGCTAAGAAC
	Reverse: 5′-AAATTT**GAATTC**TTACGCGAACGCGAAGTC
pFB-PI4KA(D1899A)	*Fragment 1:*
	Forward: 5′- CAGCTTCTGGCACACCAGTTC
	Reverse: 5′- GTTGCCGTTGTGTCTGGCCTTGATCTGCAGCAG
	*Fragment 2:*
	Forward: 5′- CTGCTGCAGATCAAGGCCAGACACAACGGCAAC
	Reverse: 5′- CGAACCCCAGAGTCCCGCTCA
pJc1/NS5A-MluI	*Fragment 1:*
	Forward: 5′-ACTGGCCATCAAGACCTTTG
	Reverse: 5′-CCCTCGAG**ACGCGT**GGGGGGCATAGAGGAGG
	*Fragment 2:*
	Forward: 5′-CCCC**ACGCGT**CTCGAGGGGGAGCCTG
	Reverse: 5′-GAGGCGCTCTTTGATGTTG
pSGR-JFH1 (NS5A-GFP)	Forward: 5′-ACGTACGCGTGGCGGTAGCATGGTGAGCAAGGGCGAG
	Reverse: 5′-TGCA**ACGCGT**ACTGCCACCCTTGTACAGCTCGTCCATGCC
pJc1(SF)	Forward: 5′GGAATT**ACGCGT**GGAGGAAGCATGGATTATAAAGATGACGATGACAAAGGGAGCGCCGCCAGCTGGAGCCATCCTCAGTTCGAGAAGGGAGGAGG
	Reverse:5′CCTTAAACGCGTGCTTCCTCCTTTCTCAAACTGTGGGTGGGACCAGCTTCCGCCTCCGCTGCCTCCGCCGCTTCCTCCTCCCTTCTCGAACTGAGGATG
	Forward flanking primer: 5′-GGAATT**ACGCGT**GGAGGAAG
	Reverse flanking primer: 5′-CCTTAA**ACGCGT**GCTTCCTC

Restriction enzymes sites are bolded.

To express T7 RNA polymerase, we added 5′ BamHI and 3′ EcoRI restriction sites flanking the T7 RNA polymerase coding sequence from pOSV-T7RP [19] by PCR (primers in Table 1). The amplified insert was digested and subcloned into BamHI/EcoRI-digested pBABE-puro retroviral vector (Addgene plasmid 1764). VSV-G pseudotyped particles were generated as described in [2]. Huh7.5.1 cells were transduced with retroviral particles in the presence of 8 µg/mL polybrene (Sigma) for 4 hr at 37°C, then selected with 2 µg/mL puromycin at 48 hr post-transduction.

For PI4KA and PI4KB silencing, T7 RNA polymerase-expressing Huh7.5.1 cells were transduced with VSV-G pseudotyped lentiviral shRNA particles derived from the pLKO.1 vector. The PI4KA shRNA sequences have been described in [2], while the PI4KB shRNA vectors (TRC numbers 0000005695 and 0000197169) were purchased from Sigma-Aldrich. After a minimum of 72 hours to allow for gene silencing, cells were plated onto poly-D-lysine coated glass coverslips. The next day, cells were transfected with pTM1(NS3-5B) or pTM1(NS3-5B/GFP) constructs using FuGENE HD (Roche, Indianapolis, IN).

### Immunofluorescence microscopy

Cells grown on poly-D-lysine coated glass coverslips were rinsed in PBS and fixed in 4% paraformaldehyde in PBS for 15 min at RT followed by quenching in 50 mM NH_4_Cl in PBS for 10 min at RT. PI4KA immunostaining was performed as described in [20]: cells were permeabilized in 0.5% Triton X-100 in PBS for 15 min at RT and rinsed with PBS; blocking and antibody incubations were performed in 3% BSA in PBS. For all other antibody immunostaining, cells were blocked and permeabilized in 0.2% saponin/10% FBS in PBS for 1 hour at RT. Primary antibody incubations were performed in blocking buffer for 1 hour at RT followed by 3 washes in PBS for 5 min each. Alexa 488 and 594-conjugated secondary antibodies (Invitrogen) were used at a dilution of 1:500 in blocking buffer. After 4 washes in PBS, coverslips were mounted with ProLong Gold with DAPI (Invitrogen) and viewed on an Olympus FluoView FV500 laser scanning confocal microscope with sequential scanning mode to limit crosstalk between fluorochromes.

### PI 4-kinase expression constructs

N-terminal myc-tagged PI4KB subcloned into pcDNA3.1/zeo(+) was a gift from Dr. Paul van Bergen en Henegouwen (Universiteit Utrecht, Netherlands). 3XFLAG-tagged PI4KA in the pFB retroviral vector has been described in [2]. The kinase-inactive PI4KA(D1899A) mutant contains a mutation in the conserved lipid kinase catalytic domain corresponding to the kinase-inactivating mutation D656A reported by Godi *et al.* for PI4KB [21] and was introduced into PI4KA using overlap extension PCR (Table 1) and the flanking restriction sites NaeI and NotI. The amplified region was completely sequenced to confirm that the D1899A mutation had been introduced without any unwanted mutations.

### Construction of epitope-tagged Jc1(SF) virus and affinity purification of NS5A(SF)

The construction of Jc1 has been described in [22]. We first introduced a unique MluI restriction site by overlap-extension PCR (Table 1) into domain III of NS5A after aa2394 (Jc1/NS5A-MluI), which had been previously shown to tolerate GFP insertion [23]. We then introduced a linker encoding a N-terminal FLAG-tag followed by a tandem Strep-tag II [24] flanked by gly-gly-ser linkers and MluI restriction sites (Table 1). Full-length Jc1(SF) RNA was prepared by XbaI linearization, T7 RNA transcription, and purification as described in [25], then introduced into Huh7.5.1 cells using a TransIT mRNA transfection kit (Mirus Bio, Madison, WI) as described in [26]. After several rounds of passaging in Huh7.5.1 cells, adapted Jc1(SF) virus was titered according to [27]. Huh7.5.1 cells were infected with Jc1(SF) or Jc1-containing cell culture supernatants at an MOI of 1 for 4 hours at 37°C. Six days post-infection, cells were harvested and lysed in 50 mM Tris pH 7.5, 150 mM NaCl, 1 mM EDTA, and 0.5% Triton X-100 with HALT protease and phosphatase inhibitors (Pierce, Rockford, IL). After centrifugation at 21,000× *g* for 15 min at 4°C to remove insoluble material, the clarified lysates were incubated with StrepTactin-Sepharose (IBA, Göttingen, Germany) for 1 hour at 4°C. Unbound material was removed by 3 washes in lysis buffer with 0.5% Triton X-100 followed by 3 washes in lysis buffer with 0.1% Triton X-100. Bound proteins were eluted with lysis buffer/0.1% Triton X-100/4 mM biotin at 4°C and then separated on SDS-PAGE for immunoblotting.

## Results

### Silencing of the PI 4-kinase PI4KA alters membranous web morphology

**Figure 1:**
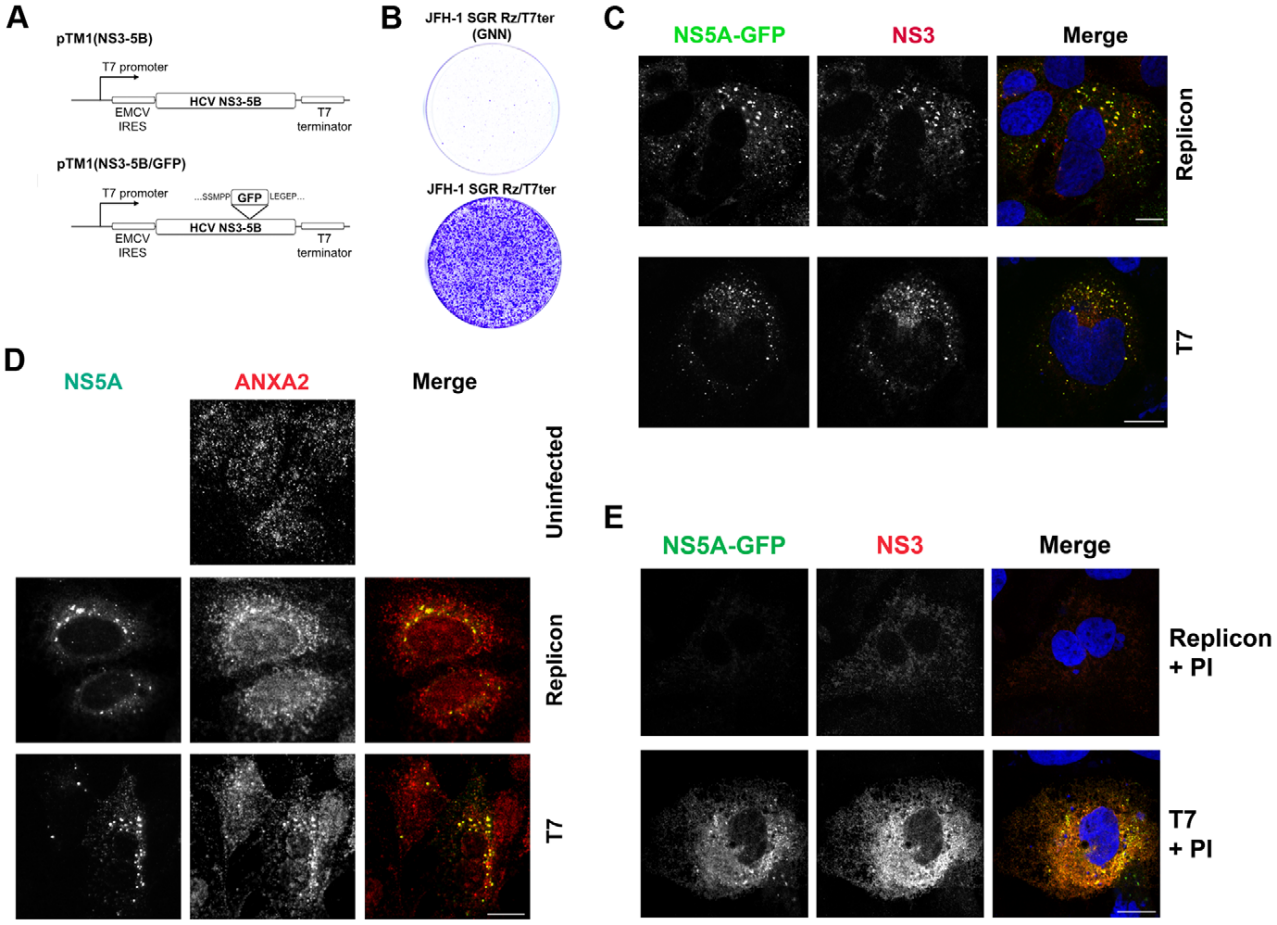
A nonreplicative system to study HCV membranous web formation. (**A**) Constructs used to express the subgenomic NS3-NS5B polyprotein under the control of a T7 promoter. The pTM1(NS3-5B/GFP) construct contains a GFP insertion within domain III of NS5A that has been previously shown to be compatible with viral replication [23]. (**B**) Recovery of replicative HCV RNA transcripts in Huh7/T7 cells stably expressing T7 RNA polymerase by a colony formation assay. Huh7/T7 cells were transfected with a DNA plasmid encoding the JFH-1 subgenomic replicon flanked by a 5′ T7 promoter and a 3′ hepatitis D virus antigenomic ribozyme and T7 terminator. The NS5B GNN mutation was introduced into this construct as a negative control. Transfected cells underwent G418 selection for three weeks and colonies were visualized by crystal violet staining. (**C**) Nonreplicative expression of NS3-5B polyprotein leads to punctate membrane structures similar in morphology to membranous webs in replicon cells. NS5A-GFP and NS3 were visualized in Huh7.5.1 hepatoma cells containing a stably replicating subgenomic HCV replicon (upper panels) compared to Huh7/T7 cells expressing the NS3-NS5B polyprotein by T7 RNA polymerase (lower panels). Nuclei were counterstained with DAPI. Bars, 10 µm. (**D**) Immunostaining of subgenomic replicon-expressing cells (upper panels) and Huh7/T7 cells expressing the HCV polyprotein (lower panels) for NS5A and for annexin A2, a host factor known to be recruited to membranous webs. Nuclei were counterstained with DAPI. Bar, 10 µm. (**E**) The effect of an NS3-4A protease inhibitor on membranous web formation is readily visualized using a nonreplicative HCV expression model. Huh7.5.1 hepatoma cells containing a HCV subgenomic replicon (upper row) or expressing HCV NS3-5B by T7 RNA polymerase (lower row) were incubated with 10 µM BILN-2061, a NS3-4A serine protease inhibitor (PI) for 48 hr and the distributions of NS3 and NS5A-GFP were visualized by confocal immunofluorescence microscopy. Nuclei were counterstained with DAPI. Bar, 10 µm.

Attempts at tetracycline-inducible expression of the full-length HCV polyprotein were unsuccessful in Huh7 or Huh7.5.1 human hepatoma cells, although they were successful in 293T human embryonic kidney cells (data not shown). We then turned to T7 RNA polymerase (T7RP)-driven expression [20], [28] of the NS3 to NS5B coding region from the genotype 2a JFH-1 strain under the control of a T7 promoter and EMCV IRES (pTM1(NS3-5B); Figure 1A). A similar construct with GFP-tagged NS5A, pTM1(NS3-5B/GFP), was generated to facilitate colocalization studies. The site of GFP tagging within domain III of NS5A has been previously shown not to significantly impair viral replication [23]. We established Huh7 cells stably expressing T7RP (Huh7/T7 cells) using a retroviral vector in order to avoid the cytotoxic effects of vaccinia virus-based T7RP expression. The EMCV IRES was used because the native HCV IRES yielded very low levels of polyprotein expression in this system (not shown); however, the resulting transcripts are incapable of RNA replication.

To demonstrate that cytoplasmically expressed T7 RNA polymerase was able to generate authentic HCV RNA transcripts in Huh7/T7 cells, we constructed a JFH-1 subgenomic replicon (bearing the HCV 5′ and 3′ NTRs) with a hepatitis D virus antigenomic ribozyme sequence immediately downstream of the HCV 3′NTR followed by a T7 terminator sequence. Transfection of the resulting JFH-1 SGR Rz/T7ter plasmid DNA into Huh7/T7 cells followed by G418 selection resulted in a high number of G418-resistant colonies (Figure 1B), whereas the JFH-1 SGR Rz/T7ter construct bearing the RNA polymerase-inactivating GDD->GNN substitutions could not form G418-resistant colonies. Transfection of the JFH-1 SGR Rz/T7ter DNA construct into Huh7.5.1 cells lacking T7 RNA polymerase also failed to produce any G418-resistant colonies (data not shown), and treatment of G418-resistant JFH1 SGR Rz/T7ter cells with 2′-C-methyladenosine, an inhibitor of the HCV NS5B RNA polymerase, markedly decreased HCV NS5A expression (not shown), consistent with active RNA replication.

As seen in Figure 1C/, Huh7T7 cells transfected with pTM1(NS3-5B/GFP) displayed a punctate cytoplasmic distribution of NS3 and NS5A-GFP that is characteristic of membranous webs [29] and that appeared similar to that seen in cells stably expressing a JFH-1 derived subgenomic replicon. No NS3 or NS5A-GFP expression was seen when Huh7 cells lacking T7RP were transfected with pTM1 expression constructs (not shown).

To further assess the degree of similarity of the membranous web-like structures generated by T7RP-driven HCV polyprotein expression to authentic membranous webs, we performed immunofluorescence staining for annexin A2, a host protein that is recruited by NS5A to HCV membranous webs [20]. As was the case in a replicon system, annexin A2 colocalized with NS5A-positive cytoplasmic puncta in the T7 expression system (Figure 1D).

We then asked whether T7RP-driven HCV expression could facilitate the identification of intermediate stages in membranous web formation. The NS3-4A serine protease releases the mature HCV nonstructural proteins by post-translational cleavage of the HCV polyprotein, and is absolutely required for viral replication. We examined the distribution of NS3 and NS5A-GFP by confocal microscopy in the T7RP expression system versus replicon cells treated with the NS3-4A protease inhibitor BILN-2061 [30]. While 48 hours of treatment with 10 µM BILN-2061 resulted in the near-complete disappearance of NS3 and NS5A-GFP expression in replicon cells, protease inhibitor treatment of Huh7/T7 cells transfected with pTM1(NS3-5B/GFP) resulted in redistribution of NS3 and NS5A-GFP to a prominent reticular pattern consistent with ER localization and the absence of a membranous web pattern (Figure 1E). We conclude that nonreplicative expression of HCV proteins can be used to study intermediate events in membranous web formation that would not be readily discerned using authentic replication systems.

**Figure 2:**
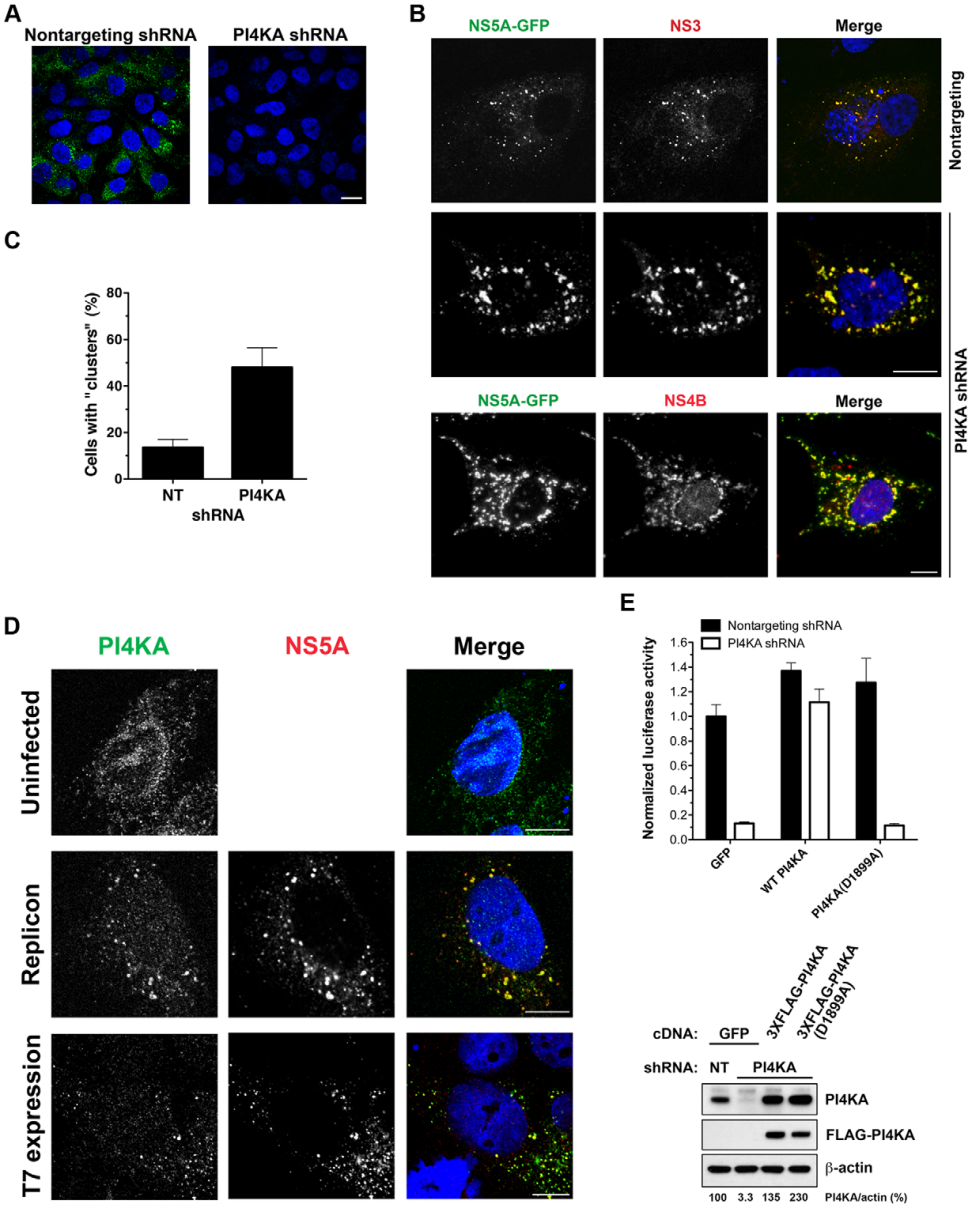
PI4KA colocalizes with HCV membranous webs and its silencing leads to aberrant web morphology. (**A**) Silencing of PI4KA leads to the disappearance of NS5A-GFP expression in HCV replicon cells. Huh7.5.1 cells expressing a subgenomic replicon were transduced with a nontargeting (left) or PI4KA-targeting (right) lentiviral shRNA vector for 5 days. HCV membranous web distribution was visualized using GFP-tagged NS5A. Bar, 20 µm. (**B**) Silencing of PI4KA leads to accumulation of membrane “clusters” in a nonreplicative model of web assembly. Huh7/T7 cells were transduced with a nontargeting (upper row) or PI4KA-targeting (lower rows) lentiviral shRNA vector and then transfected with pTM1(NS3-5B/GFP) to express the subgenomic NS3-5B polyprotein (with GFP-tagged NS5A). NS3 and NS4B were visualized by immunofluorescence staining and nuclei were counterstained with DAPI. Bars, 10 µm. (**C**) Huh7/T7 cells transduced with a nontargeting (NT) or PI4KA-targeting shRNA vector were transfected with pTM1(NS3-5B/GFP). Cells were then counted to determine the fraction with the membrane “cluster” phenotype. At least 100 cells were counted for each condition in each of three independent experiments. Values shown are means ± SD. (**D**) Colocalization of endogenous PI4KA immunofluorescence staining with NS5A in Huh7.5.1 cells expressing a subgenomic replicon (upper panels) or in Huh7/T7 cells transfected with pTM1(NS3-5B). Nuclei were counterstained with DAPI. Bars, 10 µm. (**E**) PI4KA requires kinase activity to support HCV replication. OR6 replicon cells were transduced with MMLV retroviral vectors encoding GFP, PI4KA, or a PI4KA D1899A mutant lacking kinase activity. The cells were then transduced 24 h later with lentiviral vectors encoding a nontargeting shRNA (black bars) or a shRNA targeting the PI4KA 3′UTR (white bars) to silence endogenous PI4KA. Cells were assayed 96 h after lentiviral transduction (upper panel). Values were obtained from quadruplicate wells in two independent experiments and are plotted as means ± SD. Cells in duplicate wells were lysed for immunoblotting for PI4KA, anti-FLAG, and beta-actin (lower panel) to confirm PI4KA silencing and PI4KA construct expression. PI4KA band intensities were quantitated by densitometric analysis using NIH ImageJ software and normalized to beta-actin.

Similarly, silencing of PI4KA using a lentiviral shRNA vector led to the complete disappearance of HCV nonstructural protein expression in replicon cells (Figure 2A). In contrast, PI4KA silencing followed by T7RP-driven HCV expression in Huh7.5.1 cells led to the appearance of NS5A and NS3-positive membrane “clusters” within the cytoplasm (Figures 2B and 2C) similar in morphology to those that we had previously observed in U2-OS cells [2]. Moreover, these membrane “clusters” also contained HCV NS4B, an integral membrane protein that is essential for membranous web formation (Figure 2B). These observations suggest that PI4KA is essential for the maturation of HCV-associated host membranes into replication-competent membranous webs. If true, then PI4KA should associate with HCV-associated membranes at some point during membranous web formation. Indeed, we found that endogenous PI4KA colocalized with membranous webs in a JFH1 subgenomic replicon cell line as well as in Huh7/T7 cells (Figure 2D).

To determine whether PI4KA's kinase activity is required for its ability to support HCV replication, we asked whether a kinase-dead PI4KA mutant could rescue viral replication in cells silenced for endogenous PI4KA. PI4KA(D1899A) lacking *in vitro* kinase activity [31] or wild-type PI4KA was expressed in OR6 replicon cells by retroviral transduction. The OR6 replicon contains a full-length genotype 1b HCV genome and a *Renilla* luciferase reporter gene [16]. Both PI4KA constructs lack the 3′UTR and are therefore resistant to a shRNA targeting the PI4KA 3′UTR, while the endogenous PI4KA transcript is efficiently silenced [2]. Expression of shRNA-resistant wild-type PI4KA rescued HCV replication in OR6 cells silenced for endogenous PI4KA, while the kinase-dead PI4KA(D1899A) mutant could not rescue viral replication in OR6 cells silenced for endogenous PI4KA (Figure 2E), upper panel. Silencing of endogenous PI4KA and expression of exogenous PI4KA constructs was confirmed by immunoblotting (Figure 2E), lower panel.

### PI4KA interacts with NS5A in HCV-infected cells

**Figure 3:**
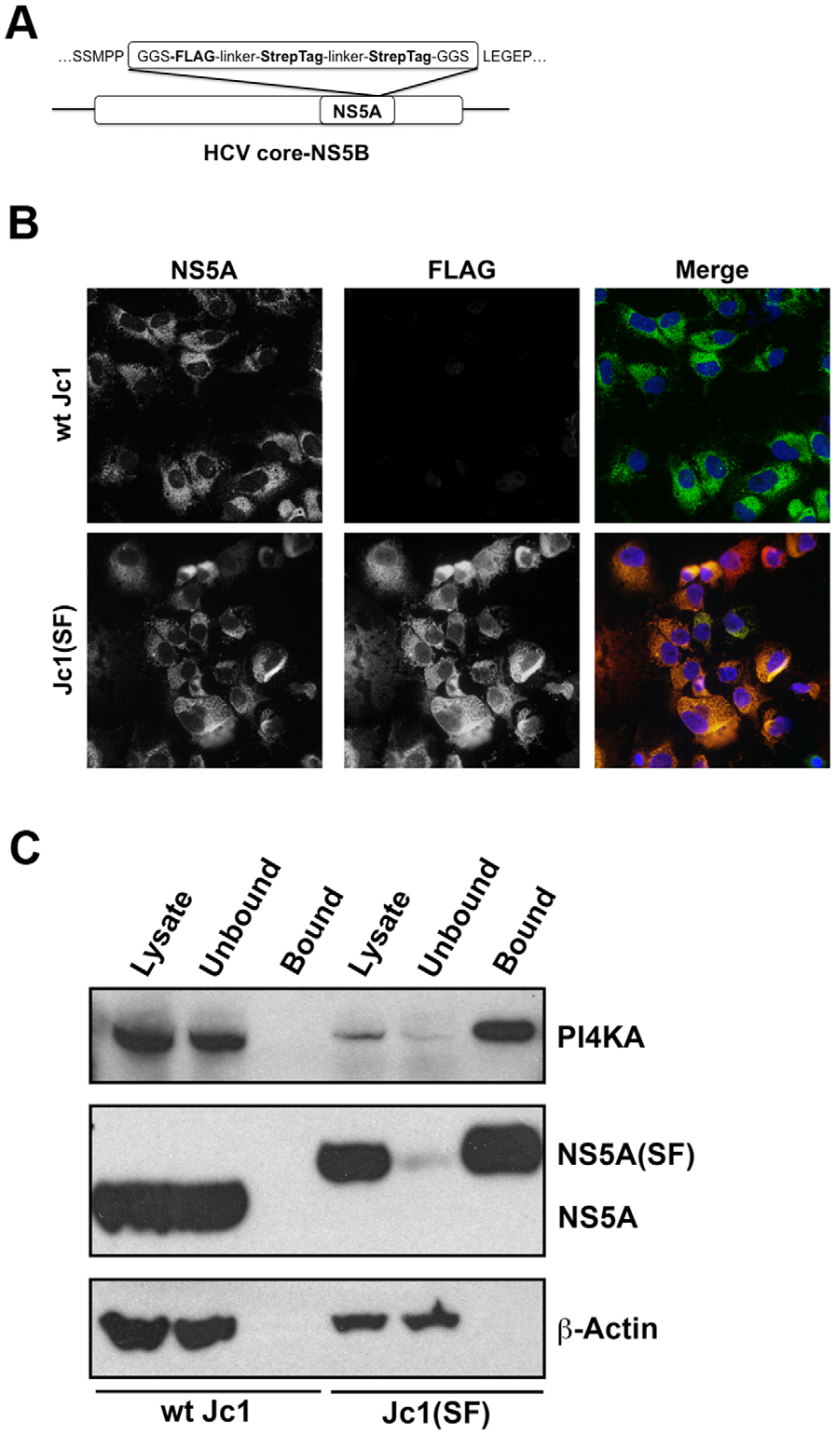
PI4KA interacts with NS5A in HCV-infected cells. (**A**) Schematic of the Jc1(SF) viral genome containing a tandem Strep-tag II and a FLAG tag in domain III of NS5A. (**B**) Productive infection of hepatoma cells with the Jc1(SF) virus. Huh7.5.1 cells were infected with wild-type Jc1 (upper panels) and culture-adapted Jc1(SF) viruses at an MOI of 1, fixed at six days after infection, and immunostained for NS5A (left panels) and FLAG (right panels). Nuclei were visualized by DAPI staining. (**C**) PI4KA associates with epitope-tagged NS5A in Jc1(SF)-infected cells. Lysates prepared from Huh7.5.1 cells infected six days prior with either wild-type Jc1 or culture-adapted Jc1(SF) were incubated with Strep-Tactin-conjugated beads. Unbound proteins were saved for analysis. After washing, NS5A(SF) and interacting proteins (“bound”) were eluted with biotin. Samples were separated by SDS-PAGE and immunoblotted for PI4KA, NS5A, and beta-actin.

Because PI4KA appears to play an essential role in HCV web formation and localizes to membranous webs, we hypothesized that it interacts with one or more viral proteins. As depicted in Figure 3A-, we inserted a small tandem affinity purification tag consisting of a tandem Streptag II and a FLAG tag [24] into domain III of NS5A at a site previously shown to be tolerant of other insertions such as GFP [23]; the backbone used was the chimeric genotype 2a Jc1 (J6/JFH1) genome [22], which is fully infectious in cell culture. The short Strep-tag II epitope tag binds to Strep-Tactin, an engineered streptavidin, and can be eluted with biotin or desthiobiotin. The resulting Jc1(SF) virus remains infectious in cell culture, and with several passages in cell culture became cell culture-adapted, reaching viral titers of >8×10^6^ TCID50/mL, comparable to wild-type Jc1 virus. Immunostaining revealed strong anti-FLAG immunoreactivity among Jc1(SF)-infected Huh7.5.1 cells, while no anti-FLAG reactivity was seen among wild-type Jc1-infected cells (Figure 3B). The epitope tag insertion was genetically stable, as evidenced by retention of FLAG immunoreactivity in all infected cells after cell culture adaptation. DAPI nuclear counterstaining also demonstrated virtually complete infection of Huh7.5.1 cells by the Jc1(SF) virus.

NS5A(SF) was isolated using Strep-Tactin-conjugated beads from lysates of Jc1(SF)-infected Huh7.5.1 cells. Wild-type Jc1 was used as a negative control. Bound NS5A(SF) and associated proteins were eluted with biotin and separated on SDS-PAGE for immunoblotting. The depleted lysate after Strep-Tactin binding was also evaluated to assess the degree of protein binding. We found that while untagged NS5A failed to bind to Strep-Tactin (Figure 3C), lane 3, NS5A(SF) was efficiently captured by the affinity matrix. Indeed, under the binding conditions used, virtually all of the NS5A(SF) was bound (Figure 3C), lanes 5 and 6. Immunoblotting revealed that PI4KA copurified with NS5A(SF) but not with untagged NS5A, consistent with a direct or indirect association between PI4KA and NS5A.

### Phosphatidylinositol 4-phosphate is enriched on HCV membranous webs

**Figure 4:**
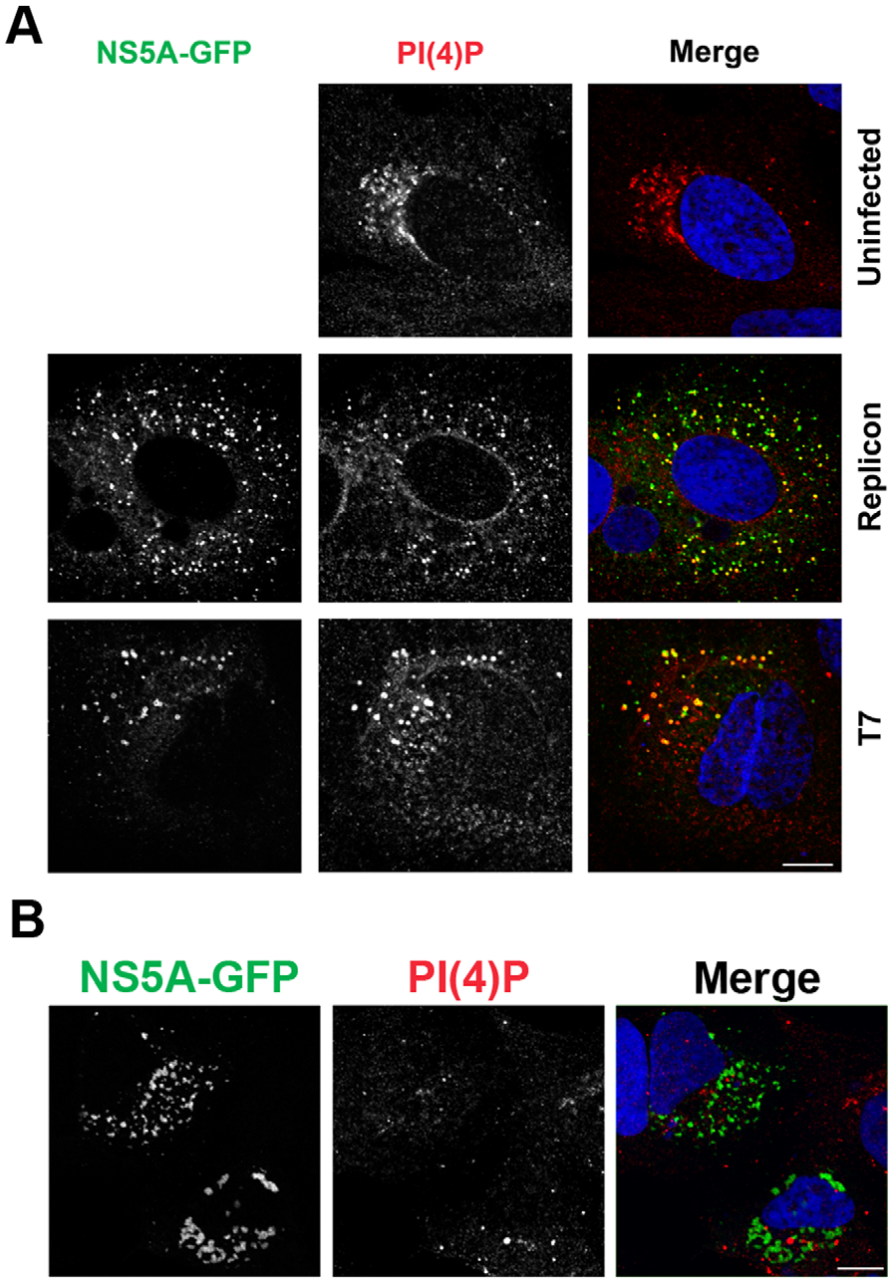
PI(4)P is enriched on HCV membranous webs. (**A**) PI(4)P immunostaining colocalizes with NS5A(GFP) at HCV membranous webs in replicon-expressing Huh7.5.1 cells (upper panels) as well as in T7-driven nonreplicative expression of NS3-5B (lower panels). Nuclei were counterstained with DAPI. Bar, 10 µm. (**B**) The membrane clusters induced by PI4KA silencing in the T7 expression model are negative for PI(4)P immunostaining. Huh7/T7 cells were silenced for PI4KA using lentiviral shRNA expression and then transfected with pTM1(NS3-5B/GFP) 24 hr prior to fixation and immunolabeling for PI(4)P. Nuclei were counterstained with DAPI. Bar, 10 µm.

Since endogenous PI4KA is found on HCV membranous webs, and because kinase-dead PI4KA does not support HCV replication, we reasoned that the product of PI4KA, phosphatidylinositol 4-phosphate, should be enriched on HCV membranous webs. Immunostaining with an anti-PI(4)P monoclonal antibody revealed enrichment of PI(4)P at HCV membranous webs in JFH1 subgenomic replicon cells as well as in the T7 expression system (Figure 4A). This observation, coupled with our previous annexin A2 and PI4KA immunostaining data, provides additional evidence that T7RP-driven HCV polyprotein expression is a good model for HCV membranous web formation.

On the other hand, the membrane clusters induced by PI4KA silencing in the T7 HCV expression system were negative for PI(4)P immunolabeling (Figure 4B), demonstrating that PI(4)P enrichment at HCV membranous webs requires PI4KA. Furthermore, this strongly suggests that the observed anti-PI(4)P immunoreactivity at HCV membranous webs is specific, and not due to nonspecific binding of the antibody to webs.

### PI4KB supports the HCV life cycle but does not localize to or generate PI(4)P at membranous webs

**Figure 5:**
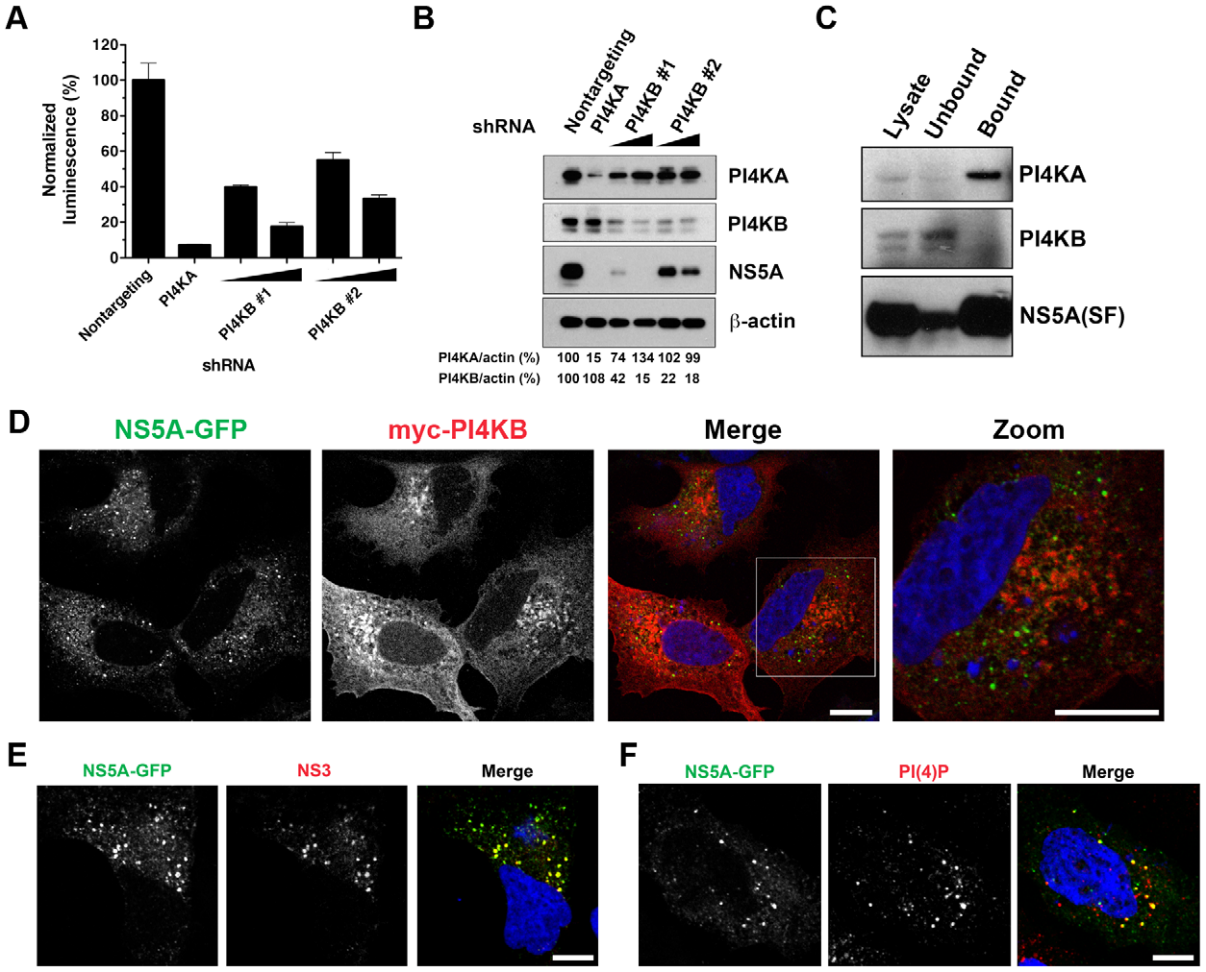
PI4KB silencing inhibits HCV infection but does not affect membranous web formation. (**A**) PI4KB silencing inhibits HCV infection. Huh7.5.1 cells were transduced with a nontargeting lentiviral shRNA construct or shRNA constructs targeting PI4KA or PI4KB. Two independent PI4KB-targeting shRNA vectors were used at two different doses. shRNA-transduced cells were infected with a Jc1/Gluc2A reporter virus encoding a *Gaussia* luciferase reporter gene. 72 hr after infection, luciferase activity in the supernatant was assayed and normalized to cells transduced with the nontargeting shRNA. Values represent means ± SD from triplicate wells in two independent experiments. (**B**) Cell lysates prepared from cells transduced with nontargeting, PI4KA, or PI4KB shRNA vectors and infected with Jc1/Gluc2A as in (A) were separated by SDS-PAGE and immunoblotted for PI4KA, PI4KB, NS5A, and beta-actin. PI4KA and PI4KB band intensities were quantitated by densitometric analysis using NIH ImageJ software and normalized to beta-actin. (**C**) PI4KB does not interact with NS5A in HCV-infected cells. Lysates prepared from Huh7.5.1 cells infected with Jc1(SF) virus were incubated with Strep-Tactin-conjugated beads. Unbound proteins were saved for analysis. After washing, NS5A(SF) and interacting proteins (“bound” lane) were eluted with biotin. Samples were separated by SDS-PAGE and immunoblotted for PI4KA, PI4KB, and NS5A. (**D**) PI4KB does not colocalize with membranous webs. Huh7.5.1 stably expressing the SGR-JFH1(NS5A-GFP) replicon were transiently transfected with a myc-PI4KB construct and immunostained with an anti-myc antibody. Bars, 10 µm. (**E**) PI4KB silencing does not alter membranous web morphology in the T7 HCV expression system. Huh7/T7 cells were silenced with the PI4KB shRNA #1 construct used in (A) and (B) prior to transfection with pTM1(NS3-5B/GFP). Cells were immunostained with anti-NS3 and NS5A-GFP was visualized by GFP fluorescence. Nuclei were counterstained with DAPI. Bar, 10 µm. (**F**) PI4KB silencing does not lead to loss of PI(4)P immunoreactivity at membranous webs. Huh7/T7 cells were silenced for PI4KB prior to transfection with pTM1(NS3-5B/GFP). Cells were immunostained with anti-PI(4)P and nuclei were counterstained with DAPI. Bar, 10 µm.

Two RNAi screens have also identified the related PI 4-kinase PI4KB as a host cofactor of HCV replication [3], [32]. We have previously shown that PI4KB overexpression does not rescue the effect of PI4KA silencing on HCV replication [2], suggesting that PI4KB supports a step in viral replication that is distinct from PI4KA. Two independent lentiviral shRNA constructs targeting PI4KB were used to silence PI4KB in Huh7.5.1 cells, followed by infection with a Jc1/Gluc2A virus encoding a secreted *Gaussia* luciferase reporter [22]. 72 hours after infection, *Gaussia* activity in the supernatant was assayed and the cells were subjected to lysis for immunoblotting. As seen in Figure 5A/, both PI4KB shRNAs did inhibit Jc1Gluc2A in a dose-dependent manner, though not as strongly as with PI4KA silencing. Immunoblotting revealed that PI4KB was depleted by both PI4KB-specific shRNAs and showed that PI4KB silencing was associated with a dose-dependent decrease in NS5A protein levels (Figure 5B).

Furthermore, no association between endogenous PI4KB and epitope-tagged NS5A was identified in Jc1(SF)-infected cells (Figure 5C) under the conditions used to demonstrate the association of PI4KA with NS5A.

At steady-state, PI4KB predominantly localizes to the Golgi [21], and is recruited to enterovirus-induced host membrane alterations [10]. In SGR-JFH1(NS5A-GFP) replicon cells transfected with a myc-PI4KB expression construct, myc-PI4KB was found to be distributed in a pattern consistent with a Golgi membrane localization, and did not colocalize with HCV membranous webs (Figure 5D). Furthermore, silencing of PI4KB did not cause any apparent alterations in web morphology in the T7 expression system (Figure 5E), nor did it lead to the loss of PI(4)P immunostaining on membranous webs (Figure 5F). Therefore, the enrichment of PI(4)P at membranous webs appears to be due to the activity of PI4KA and does not require PI4KB. We conclude that while PI4KB may support HCV replication, its function appears to be different from that of PI4KA.

### PI4KA silencing-induced membrane clustering depends on HCV polyprotein cleavage but does not require integrity of the ER-Golgi secretory pathway

**Figure 6:**
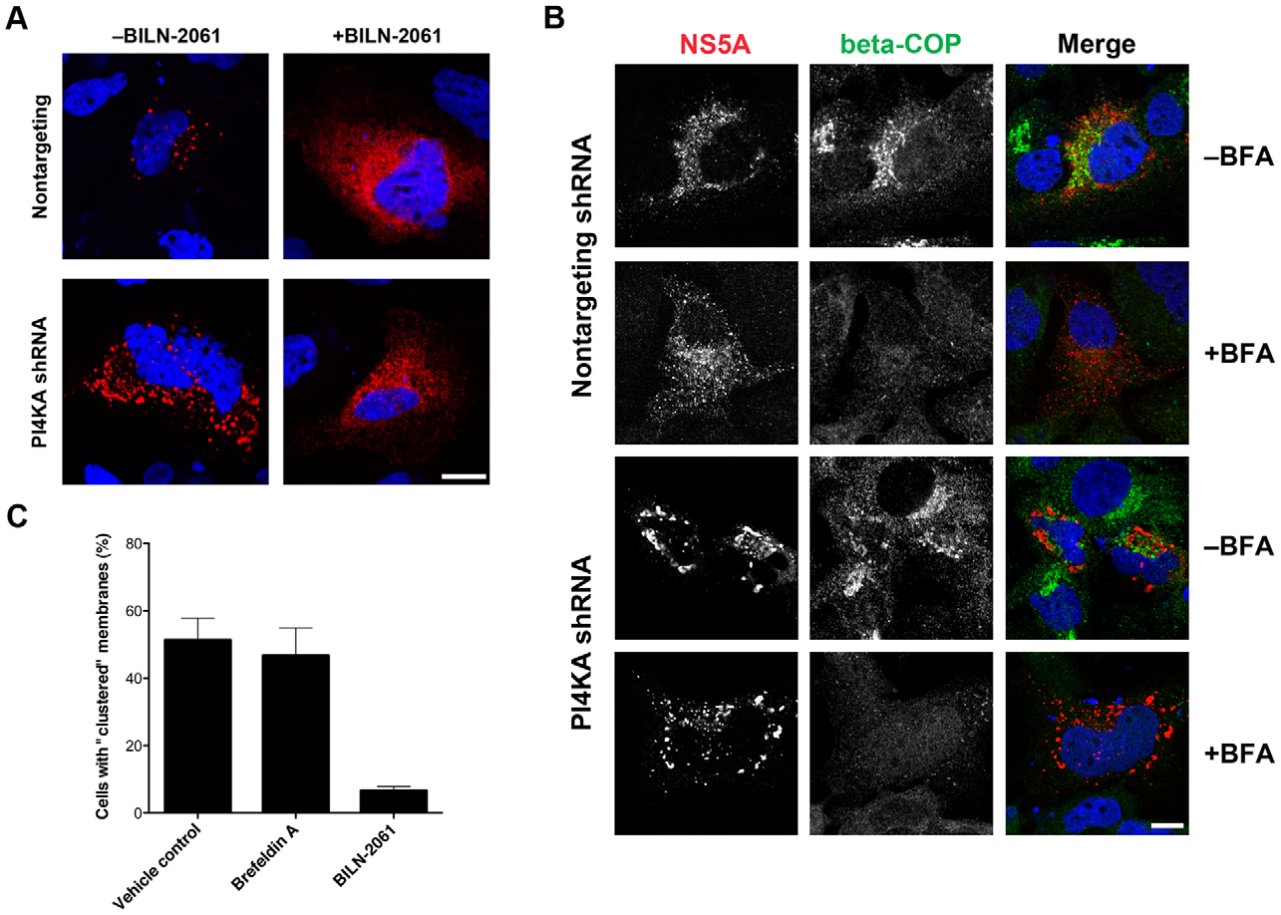
HCV-induced membrane clustering in PI4KA-silenced cells requires NS3-4A protease activity but does not require host secretory pathway integrity. (**A**) Formation of membrane “clusters” in PI4KA-silenced cells expressing HCV proteins requires HCV polyprotein cleavage. Huh7/T7 cells were transduced with a nontargeting (upper panels) or a PI4KA-targeting (lower panels) shRNA vector prior to pTM1(NS3-5B) transfection. Cells were treated with 0.5% DMSO (left panels) or with 10 µM BILN-2061 (right panels) for 24 hours prior to fixation and immunostaining for NS5A. Nuclei were counterstained with DAPI. Bar, 10 µm. (**B**) Formation of membrane “clusters” in PI4KA-silenced cells expressing HCV proteins does not require integrity of the host secretory pathway. Huh7/T7 cells were transduced with a nontargeting (upper panels) or a PI4KA-targeting (lower panels) shRNA vector prior to pTM1(NS3-5B) transfection. Upon transfection, cells were treated with 0.1% ethanol or with 100 ng/mL BFA for 24 hours prior to fixation and immunostaining for NS5A and beta-COP (to demonstrate COPI coatomer dispersal by BFA). Nuclei were counterstained with DAPI. Bar, 10 µm. (**C**) Cells from the experiments shown in panels (A) and (B) were counted to determine the fraction with the membrane “cluster” phenotype. A minimum of 100 cells were counted for each condition in each of two independent experiments. Values shown are means ± SD.

The ability to study HCV membranous web assembly without ongoing viral replication allows us to study interactions between two events or host genes that are required for HCV web assembly. As proof-of-concept, we first hypothesized that HCV nonstructural protein cleavage by the NS3-4A viral protease is required for the accumulation of NS5A-positive membrane clusters in PI4KA-silenced cells. T7-driven expression of HCV NS3-5B in PI4KA silenced cells led to the formation of membrane clusters (Figure 6A), as expected. However, addition of the NS3-4A protease inhibitor BILN-2061 to PI4KA-silenced cells led to inhibition of membrane cluster formation (Figures 6A and 6C), demonstrating that formation of this membranous web intermediate requires posttranslational cleavage of the viral nonstructural proteins [33].

We then asked whether membrane cluster formation in PI4KA-silenced cells requires an intact ER-Golgi secretory pathway. Brefeldin A (BFA) inhibits ADP ribosylation factors such as ARF1 by targeting guanine nucleotide exchange factors such as GBF1. ARF1 inhibition, in turn, disrupts early secretory membrane trafficking by preventing COPI coat assembly. ARF1, GBF1, and COPI coatomer have all been shown to be cofactors of HCV replication [2], [34], [35]. BFA inhibits HCV replication when added at the time of or shortly after infection but is less effective at inhibiting replication after infection has been established [2], suggesting that GBF1 and ARF1 act early in the viral lifecycle. We initially hypothesized that these factors could interfere with membranous web formation. We found, however, that BFA treatment at 100 ng/mL, a dose sufficient to inhibit HCV replication [2] and cause dissociation of beta-COP from the Golgi, failed to prevent the formation of NS5A-positive membrane clusters in PI4KA-silenced cells (Figures 6B and 6C) or the formation of NS5A-positive puncta in nontargeting shRNA control cells. These findings suggest that GBF1, ARF1, and COPI act downstream of PI4KA in the HCV lifecycle and that membrane cluster formation does not require an intact early secretory pathway.

## Discussion

It has been known for decades that a number of viruses induce membrane alterations within the infected host cell [36]. This appears to be universally true among positive-sense RNA viruses such as HCV, and has been postulated to shield viral RNA and other pathogen-associated molecular patterns from host innate immune recognition and defenses. Electron tomographic studies of dengue-infected cells have demonstrated the induction of a complex network of ER-derived interconnected membranes of varying morphologies [37]. However, our understanding of the molecular mechanisms that direct these membrane alterations has lagged behind their morphologic characterization. One reason for this has been our incomplete understanding of the cellular factors that are exploited by HCV for membranous web assembly. Recent RNAi screens have identified previously unknown host dependency factors, such as PI4KA, that are essential for HCV replication [2], [3], [5],[32],[38]. At least some of these factors may be important for web formation. Another reason has been the dependence of HCV polyprotein expression on active viral RNA replication. At the long timescales needed for RNAi experiments, silencing of factors required for the viral lifecycle leads to the loss of new membranous web formation in models that depend on active viral RNA replication, such as replicons or cell culture-infectious HCV. In order to circumvent this limitation, nonreplicative systems of HCV polyprotein expression and web formation must be used.

In our previous work, we found that PI4KA silencing led to the formation of NS5A-positive membrane clusters in a U2-OS osteosarcoma cell line model of inducible HCV polyprotein expression [2], [14]. However, studies of HCV membranous web assembly are better performed in a more physiologically relevant hepatocyte cell line. In this study, we used a T7 RNA polymerase-based expression system [20], [28] in Huh7 human hepatoma cells to study the roles of PI4KA and PI4KB on HCV membranous web assembly. T7RP-driven expression of the HCV NS3-5B polyprotein results in the generation of structures that are similar to authentic HCV membranous webs based on their morphology, subcellular distribution, and the presence of common viral and host markers (NS5A, NS3, annexin A2, PI(4)P, and PI4KA).

During the preparation of this manuscript, two other groups reported their work on PI4KA and HCV replication [6], [39]. Reiss et al. found colocalization of PI(4)P and PI4KA, but not PI4KB, with the HCV membranous web and showed that nonreplicative expression of HCV nonstructural proteins in PI4KA-silenced cells induced clusters of small double-membrane vesicles. Both groups found that cellular PI(4)P levels were increased in HCV-infected cells in a PI4KA-dependent manner, and that NS5A stimulated PI4KA lipid kinase activity *in vitro*. Our findings substantially agree with both of these studies. In addition, we propose that the HCV-induced membrane “clusters” seen in PI4KA silenced cells represent accumulation of a specific intermediate in HCV membranous web formation. The generation of these clusters from their cellular membrane(s) of origin does not require PI4KA, thus defining an early stage in host membrane rearrangement that is PI4KA and PI(4)P-independent. In addition, we show that formation of the HCV-induced membrane clusters seen in PI4KA silenced cells requires NS3-4A serine protease activity, suggesting that cluster formation requires the specific activities of one or more viral nonstructural proteins and is not merely a nonspecific effect of the T7RP expression system. Finally, formation of this HCV membranous web intermediate is resistant to BFA treatment and is therefore not dependent on integrity of the ER-Golgi secretory pathway. This result is consistent with the model that the HCV membranous web arises directly from the ER; if the web were produced from a post-ER membrane compartment, we would expect cluster formation to be BFA-sensitive. The mechanism by which ARF1, GBF1, and COPI support HCV replication remains to be elucidated. Goueslain *et al.* have shown that BFA treatment of HCV-infected Huh7 cells results in ultrastructural disorganization of membranous web structure, but BFA treatment does not prevent the appearance of membranous web-like structures in U2-OS cells induced to express the HCV polyprotein [34]. Our findings that membrane cluster formation is insensitive to BFA treatment agrees with their conclusion that BFA-sensitive factors are not required for web morphogenesis, but perhaps for web activity.

The association of PI4KA with HCV-induced membrane alterations is likely to stimulate the local generation of PI(4)P. Of note, while PI4KB does not appear to be recruited to HCV membranous webs, it is associated with enteroviral membrane alterations [10], suggesting that local PI(4)P production may be necessary for the replication of a number of positive-sense RNA viruses. It remains to be determined whether other positive-sense viruses hijack other PI 4-kinase family members for their own replication. If so, this would raise the possibility that strategies to disrupt viral-induced PI(4)P accumulation might have activity against multiple positive-sense RNA viruses.

Subsequent steps in membranous web formation have yet to be defined, but are likely to involve the recruitment of host and/or viral PI(4)P-binding protein(s). For instance, the poliovirus 3D RNA polymerase binds to PI(4)P in a protein-lipid overlay assay [10], suggesting a mechanism by which viral components might be recruited and/or retained at replication sites. It remains to be determined whether any HCV proteins interact directly with PI(4)P. Alternatively or additionally, host PI(4)P binding proteins could be recruited to the maturing HCV membranous web. One candidate is oxysterol-binding protein (OSBP), which binds to PI(4)P and has also been shown to regulate HCV particle assembly [40]. Future studies will seek to identify the effectors of PI4KA and PI(4)P at the HCV membranous web.

One limitation of this work is the identification of only two intermediates in web formation: the relatively trivial ER retention pattern shown by NS3-4A protease inhibition, and the PI(4)P-negative membrane clusters revealed by PI4KA inhibition. Inhibiting other known host factors of HCV replication in nonreplicative web formation systems may lead to the identification of additional intermediate stages of HCV web formation. In addition, we will need to move beyond morphological descriptions of membranous webs and their intermediates, as these will be reliable only for extreme phenotypes. More sensitive and specific techniques to dissect web assembly mechanisms might include the identification of additional reliable markers of the web and its intermediates (such as PI(4)P), and the characterization of their biochemical and biophysical properties.
